# Key Anti-Fibrosis Associated Long Noncoding RNAs Identified in Human Hepatic Stellate Cell via Transcriptome Sequencing Analysis

**DOI:** 10.3390/ijms19030675

**Published:** 2018-02-27

**Authors:** Xian-Qian Li, Zhen-Xing Ren, Ke Li, Jing-Jing Huang, Zi-Tong Huang, Tian-Ran Zhou, Hong-Ying Cao, Feng-Xue Zhang, Bo Tan

**Affiliations:** 1 The Research Center for Integrative Medicine, School of Fundamental Medical Sciences, Guangzhou University of Chinese Medicine, Guangzhou 510006, China; lxianqian0507@gmail.com (X.-Q.L.); rzhenxing2016@gmail.com (Z.-X.R.); a15813359410@gmail.com (K.L.); tannyhy@gmail.com (J.-J.H.); zitong531@gmail.com (Z.-T.H.); tianran531@gmail.com (T.-R.Z.); zhangfengxue@gzucm.edu.cn (F.-X.Z.); 2 School of Chinese Materia Medical, Guangzhou University of Chinese Medicine, Guangzhou 510006, China; hycao@gzucm.edu.cn

**Keywords:** RNA-sequencing, lncRNA, hepatic fibrosis, human hepatic stellate cell, fibroblast growth factor 2 (*FGF2*), netrin-4 (*NTN4*)

## Abstract

Hepatic fibrosis is the main pathological basis for chronic cirrhosis, and activated hepatic stellate cells (HSCs) are the primary cells involved in liver fibrosis. Our study analyzed anti-fibrosis long noncoding RNAs (lncRNAs) in activated human HSCs (hHSCs). We performed RNA sequencing (RNA-seq) and bioinformatics analysis to determine whether lncRNA expression profile changes between hHSCs activation and quiescence. Eight differentially expressed (DE) lncRNAs and three pairs of co-expression lncRNAs-mRNAs were verified by quantitative Real-Time Polymerase Chain Reaction (qRT-PCR). A total of 34146 DE lncRNAs were identified in this study. Via gene ontology (GO) and Kyoto Encyclopedia of Genes and Genomes (KEGG) analyses, we found several DE lncRNAs regulated hHSC activation by participating in DNA bending/packaging complex, growth factor binding and the Hippo signaling pathway (*p* < 0.05). With lncRNA–mRNA co-expression analysis, three lncRNAs were identified to be associated with connective tissue growth factor (*CTGF*), fibroblast growth factor 2 (*FGF2*) and netrin-4 (*NTN4*). The quantitative Real-Time Polymerase Chain Reaction (qRT-PCR) results of the eight DE lncRNAs and three pairs of co-expression lncRNAs–mRNAs were consistent with the RNA-seq data and previous reports. Several lncRNAs may serve as potential targets to reverse the progression of liver fibrosis. This study provides a first insight into lncRNA expression profile changes associated with activated human HSCs.

## 1. Introduction

Liver fibrosis primarily refer to the deposition of extracellular matrix deposition and hyperplasia of abnormal fibrous tissue, which characterizes the early stage of cirrhosis. Hepatic stellate cells (HSCs) are non-parenchymal cells, located in the Disse gap. The HSC transformation from quiescence to activation is the core of liver fibrosis [1]. HSCs are also activated to produce extracellular matrix proteins [2], and thereby genes, such as α2 smooth muscle actin (α-SMA), actin, alpha 2 (*ACTA2*) [3], collagen type I alpha 1 chain (*COL1A1*) [4], lysyl oxidase (*LOX*) [5] and lysyl oxidase like 2 (*LOXL2*) [6], which are all involved in the dynamic process of HSC myofibroblasts.

HSCs are activated and transformed by multiple factors and pathways [7]. As previously described, both *FGF2* [8] and fibroblast growth factor receptor 2 (FGFR2) [9] were considered as cell growth factors that protect the liver from fibrosis. Classical signaling pathways and molecular functions, such as the inhibition of HSC collagen secretion and the activation of the transforming growth factor beta (TGF-β)/Smad and Peroxisome Proliferator-activated Receptor (PPAR) pathways also occur. Meanwhile, the Hippo pathway involved in hepatic fibrosis development is a newly researched mechanism [10,11,12]. Previous studies confirmed that Hippo in the liver can regulate organ size and cell fate [11]. However, it remains unknown whether the lncRNA mediate *FGF2*, *FGFR2* or participated in the Hippo pathway to regulate liver fibrosis.

Long noncoding RNA (lncRNA) are one of noncoding RNA families, with a transcription length of more than 200 nucleotides (nt). It is structurally similar to mRNA; however, it is incapable of protein coding [13,14]. Numerous works demonstrated that increasing numbers of lncRNAs play critical regulatory roles not only in human disease processes, but also as novel therapeutic targets in cancer [15,16,17]. Genome-wide RNA sequencing (RNA-seq) was widely used in basic research to identify key lncRNAs that regulate diseases [18,19]. It has been reported that 12 lncRNAs and 155 mRNAs were identified to be upregulated in activated rat HSCs by RNA-seq analysis, and in addition, the potential role of upregulated lncRNA NONRATTO13819.2 and Lox in extracellular matrix (ECM) remodeling during activation [20]. Some 3600 lncRNAs were identified in human HSC myofibroblasts in combination with RNA-seq and chromatin immunoprecipitation sequencing (ChIP-seq). The lncRNAs that were directly regulated by TGF-β signaling were enriched in super enhancers [21]. lncRNA *NEAT1* promotes the progression of hepatic fibrosis by regulating Kruppel-like factor 6 [22], whereas the *lnc-LFAR1*, which was thought to be a liver-enriched lncRNA, accelerates hepatic fibrosis via targeted TGFβ and the Notch pathway [23]. However, the number of lncRNAs identified in hHSC myofibroblasts is limited. Hence, further studying the complex biological functions of lncRNAs in activation and quiescence of hHSCs is difficult. In this study, we performed RNA-seq and bioinformatics analysis to identify several key anti-fibrosis associated lncRNAs in hHSCs activation and quiescence.

## 2. Results

### 2.1. VPA-Mediated Induction of hHSC Myofibroblasts Conversion into an Inactive Phenotype Subsection

**Figure 1:**
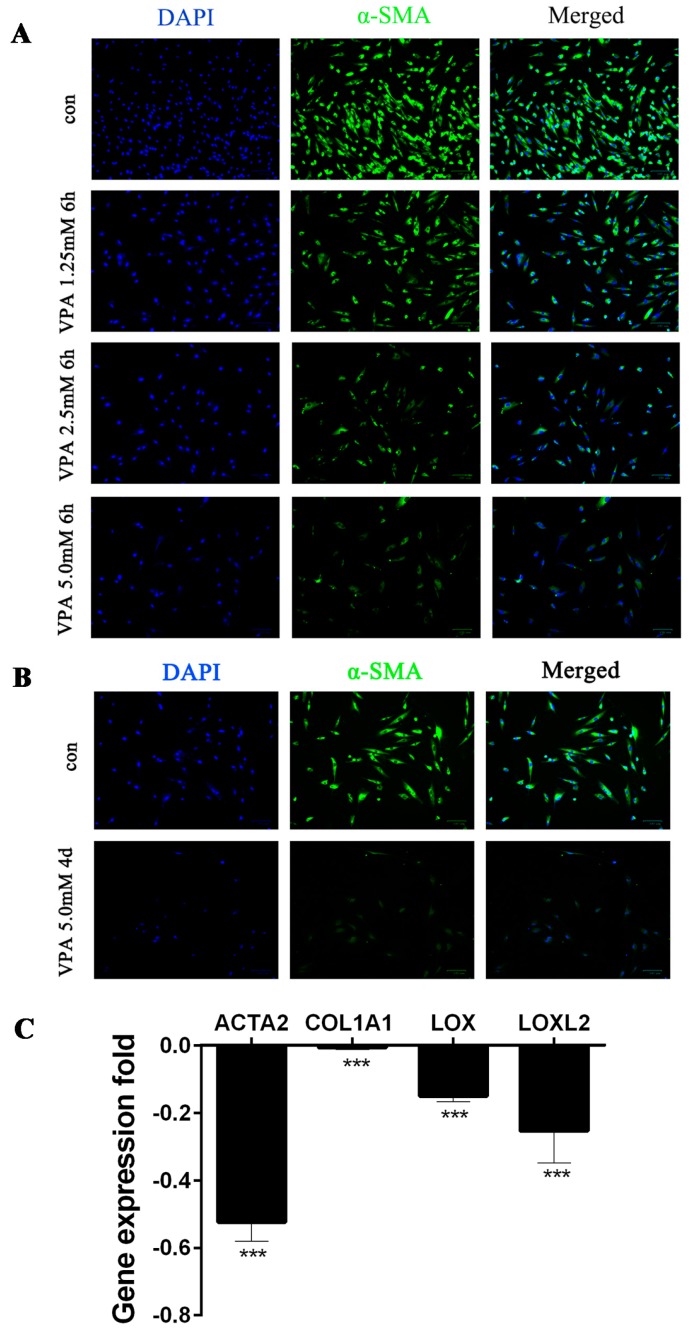
Valproic acid (VPA)-mediated induction of human hepatic stellate cell (hHSC) myofibroblast conversion into an inactive phenotype. Immunofluorescence microscopy for α2 smooth muscle actin (α-SMA) expression in hHSC myofibroblasts treated with VPA (1.25, 2.5 and 5.0 mM) for 6 h and 4 days (**A**,**B**). hHSCs were cultured in dulbecco’s modified eagle medium (DMEM) containing 5% fetal bovine serum (FBS) and 1% Penicillin/Streptomycin (P/S) in the presence or absence of VPA; nuclei were stained with 4′,6-diamidino-2-phenylindole (DAPI) (blue), and α-SMA is indicated as green; magnification: ×100 μm. (**C**) Alpha 2 (*ACTA2*), collagen type I alpha 1 chain (*COL1A1*), lysyl oxidase (*LOX*) and lysyl oxidase like 2 (*LOXL2*) mRNA expression folds of hHSCs treated with 5.0 mM VPA for 4 days were normalized against activated hHSCs control (con). The samples were normalized using ribosomal protein S18 (18S), the error bar represents standard deviation, and this experiment is representative of three biological replicates. (*** *p* < 0.001).

Valproic acid (VPA) is a novel class of histone deacetylase (HDAC) inhibitor. hHSCs were treated with VPA (1.25, 2.5 and 5.0 mM) for 6 h and 4 days (d), and the expression levels of α-SMA were determined by immunofluorescence (Figure 1A,B). Immunofluorescence microscopy revealed that the α-SMA expression levels in VPA (1.25, 2.5 and 5.0 mM) for the 6-h group were decreased in contrast to those of the control (con) group (Figure 1A). However, the α-SMA expression in the VPA 5.0 mM 4-day group revealed more significantly decrease than any VPA group (1.25, 2.5 and 5.0 mM) for 6 h and the con (Figure 1B). The mRNA expression levels of *ACTA2*, *COL1A1*, *LOX* and *LOXL2* in hHSC myofibroblasts (cultured in the absence or presence of 5.0 mM VPA for 4 days) were quantified using qPCR. mRNA expression levels were more significantly suppressed relative to that in Figure 1C. Thus, 5.0 mM VPA treatment for 4 days was selected for hHSC myofibroblasts induction into an inactive phenotype. The VPA group described below represents VPA 5.0 mM 4 days (quiescent hHSC), while the con group represents activated hHSC.

### 2.2. Overview of RNA-Seq in hHSCs Myofibroblasts

**Figure 2:**
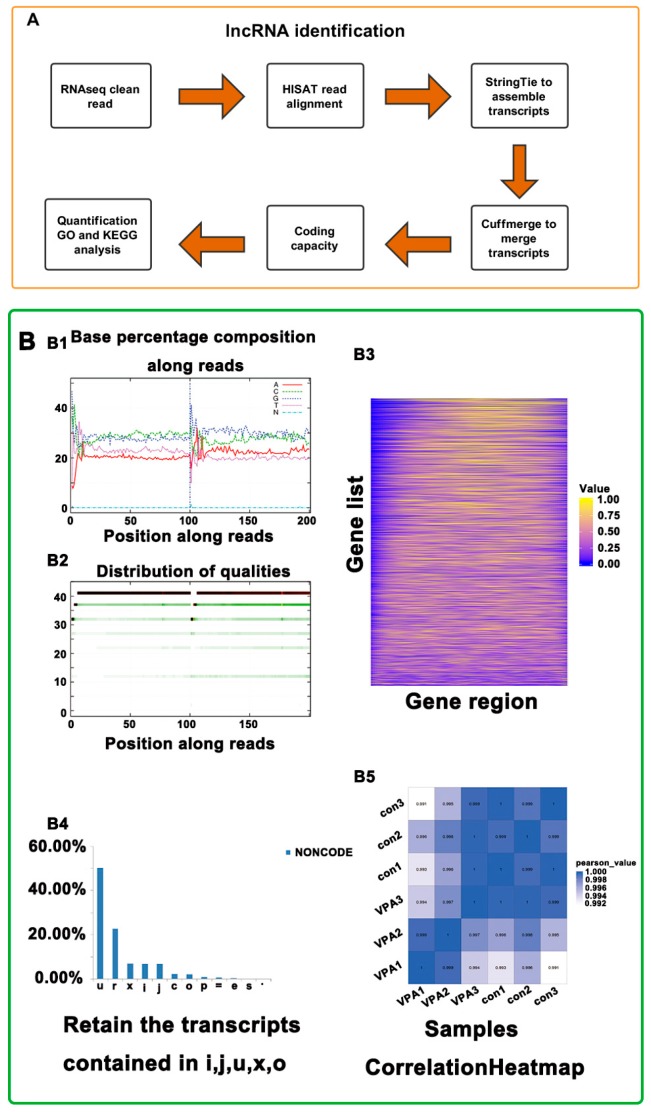
Overview of sequencing RNA (RNA-seq) in hHSC myofibroblasts. Firstly, we employed hierarchical indexing for spliced alignment of transcripts (HISAT) to align clean reads to the human reference genome (hg19/grch37). Secondly, we assembled the transcripts by StringTie for each sample and then used Cufflinks to merge the assembled transcripts. Thirdly, we combined three computational methods, CPC/txCdsPredict/CNCI/Pfam, to distinguish the mRNAs and long noncoding RNAs (lncRNAs) in the assembled transcripts. We applied Bowrie2 to compare clean reads to the reference sequence and then used RNA-Seq by Expectation Maximization (RSEM) to calculate for the expression levels of the genes and transcripts (**A**). To ensure the reliability of further data analysis at each step of data acquisition or analysis process, we applied a specific filter and check, including Base msaa distribution, sample correlation and sequencing depth, to monitor the data quality (**B**), and Figure includes Base distribution (**B1**), base mass distribution (**B2**), gene coverage map (**B3**), lncRNA classification chart from NONCODE (**B4**) and B5 is sample (con1, con2, con3 and VPA1, VPA2, VPA3 biological duplication) correlation (**B5**).

An overview of the analysis pipeline is shown in Figure 2. The average scores for Q20ofFq1 and Q30ofFq1 were 99.31% and 97.68%, respectively (Table S1). The results indicated that the sequence data quality and samples reproducibility were high. Moreover, average totals of 189,956,757.3 and 178,038,730 raw reads were generated for the con and VPA groups, respectively. The raw reads were then filtered, resulting in 175,359,348 and 164,659,836.7 clean reads on average for the con and VPA groups, respectively. The clean reads with removed rRNA were mapped to the human reference genome by using hierarchical indexing for spliced alignment of transcripts (HISAT). More than 77% of the average reads were mapped to the human reference genome, and more than 68% of the average reads were uniquely mapped to the genome (Table S1).

### 2.3. Identification of lncRNA and mRNA in hHSC Myofibroblasts

**Figure 3:**
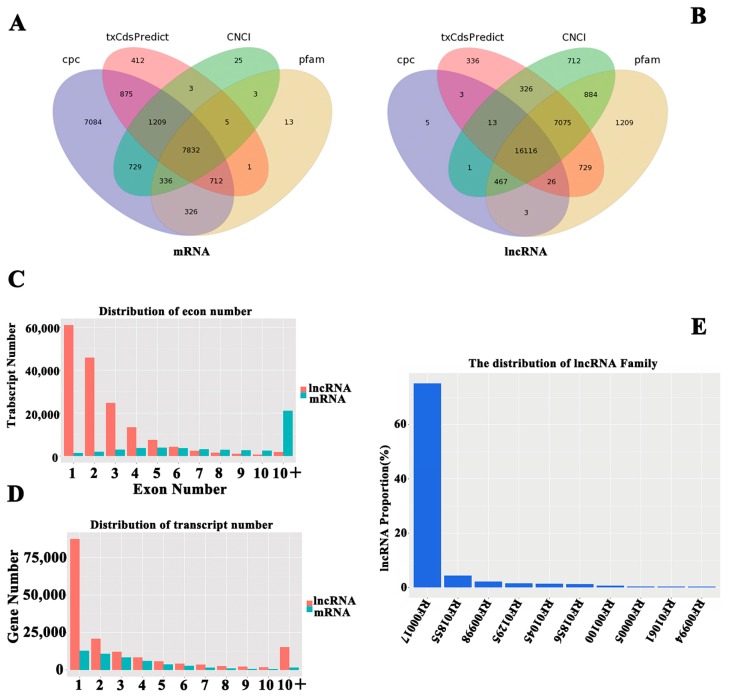
Identification of lncRNAs and mRNAs in hHSC myofibroblasts. Three computational methods, CPC/txCdsPredict/CNCI/Pfam, were combined to distinguish the mRNAs and lncRNAs in the assembled transcripts. The venn diagram illustrates the distribution of the lncRNAs and mRNAs in the CPC/txCdsPredict/CNCI/Pfam four software databases, including the distribution of the mRNA (**A**) and lncRNA (**B**). (**C**) Exon contents of the lncRNAs and mRNAs. (**D**) Transcript number distribution of the lncRNA and mRNA genes. (**E**) lncRNAs and mRNAs lengths.

An average of 24,169 known lncRNAs, 7469 known mRNAs, 3922 novel lncRNAs and 2244 novel mRNAs were identified in each sample (Table S2). The prediction of lncRNA coding ability in hHSCs is presented in Figure 2A,B. The number of transcripts in the exon analysis suggested that the lncRNAs and mRNAs have a concentrated number of exons ranging from 1–3 and 10+, whereas the average number of mRNA exons was greater than that of the lncRNA exons (Figure 3C). The transcript number distributions of the lncRNA and mRNA genes indicated that most of the lncRNA and mRNA genes contained 1–5 transcripts, and the largest proportion of the lncRNA and mRNA genes contained only one transcript (Figure 3D). The distribution patterns of the lncRNAs and mRNAs in hHSC myofibroblasts were similar. Next, INFERNAL [24] was employed to compare lncRNAs with the Rfam database for annotations and lncRNA classification (RF00017, RF01855, RF00998, RF01295, RF01045, RF01856, RF00100, RF00005, RF01061 and RF00994). Most of the lncRNAs in the hHSC myofibroblasts were noted belong to the RF00017 family ID (Figure 3E).

### 2.4. Profiling of Differentially Expressed (DE) lncRNA and mRNA in Activated hHSCs and Quiescent hHSCs

**Figure 4:**
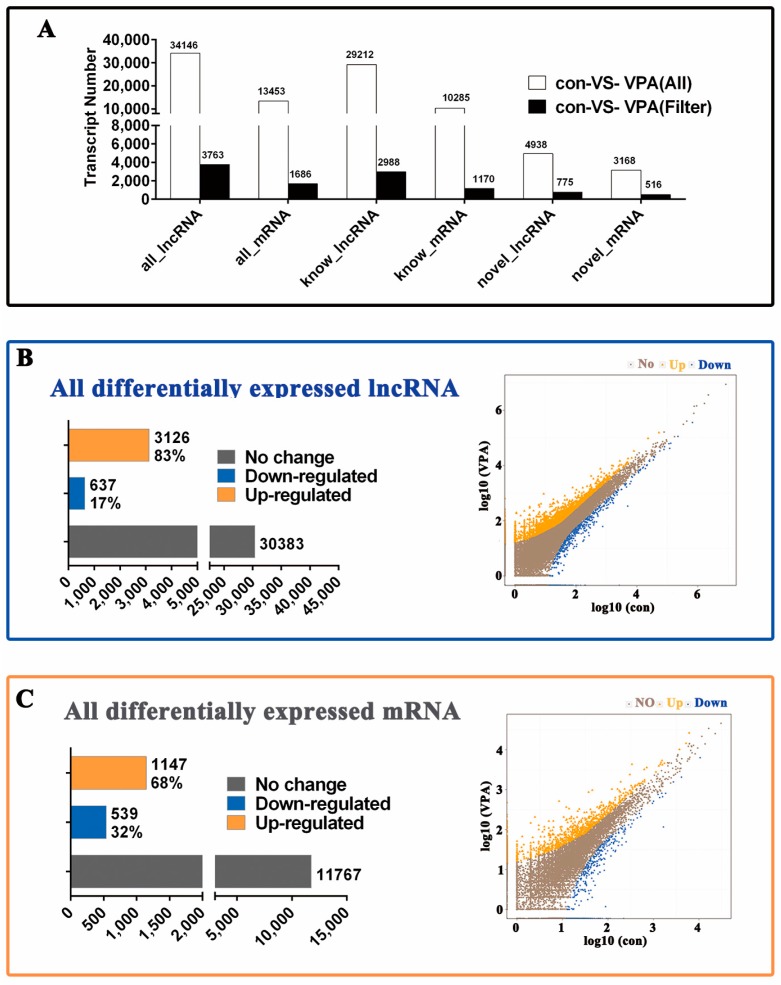
Profiling of the differentially expressed (DE) lncRNAs and mRNAs in the hHSC myofibroblasts between the activated hHSCs (con) and quiescent hHSCs (VPA). (**A**) Histograms represent the significant and nonsignificant differences in transcripts between the con (activated hHSC) and VPA (quiescent hHSC), con-VS-VPA (All) is the significant differences, whereas the con-VS-VPA (Filter) represents the significant differences only. (**B**,**C**) Histograms and Scatter plot of the co-expressed transcripts between con and VPA. (**B**,**C**) represent the lncRNAs and mRNAs, respectively. Blue denotes downregulated RNA transcripts, orange denotes the upregulated RNA transcripts, and brown denotes the nonregulated RNA transcripts in VPA (quiescent hHSC) compared with con (activated hHSC).

A total of 34,146 DE lncRNAs, including 29,212 known DE lncRNAs and 4938 novel DE lncRNAs, and 13,453 DE mRNAs, including 10,285 known DE mRNAs and 3168 novel DE mRNAs were obtained. Significant difference analysis was performed for the screening of transcripts without significant changes in expression levels. Afterwards, 3763 significant (Filter) DE lncRNAs, including 2988 known significant (Filter) DE lncRNAs and 775 novel significant (Filter) DE lncRNAs, and 1686 significant (Filter) DE mRNAs, including 1170 known significant (Filter) DE mRNAs and 516 novel significant (Filter) DE mRNAs, are achieved (Figure 4A).

When false discovery rate (FDR) ≤0.001 and absolute log2 ratio >1, 3126 upregulated and 637 downregulated lncRNA DE transcripts were present in 3763 significantly DE lncRNAs (Figure 4B), and 1147 upregulated and 539 downregulated lncRNA DE transcripts in 1686 significantly DE mRNAs (Figure 4C). These results suggest that The DE lncRNA transcripts were relatively greater in number than the mRNA transcripts in the con (activated hHSC) and VPA (quiescent hHSC). Furthermore, most of the lncRNA (about 83%) and mRNA (about 68%) were upregulated significantly in the DE lncRNAs and mRNAs, in contrast to those in the VPA (quiescent hHSC) group.

### 2.5. GO and KEGG Enrichment Analysis of DE lncRNA Target Genes

**Figure 5:**
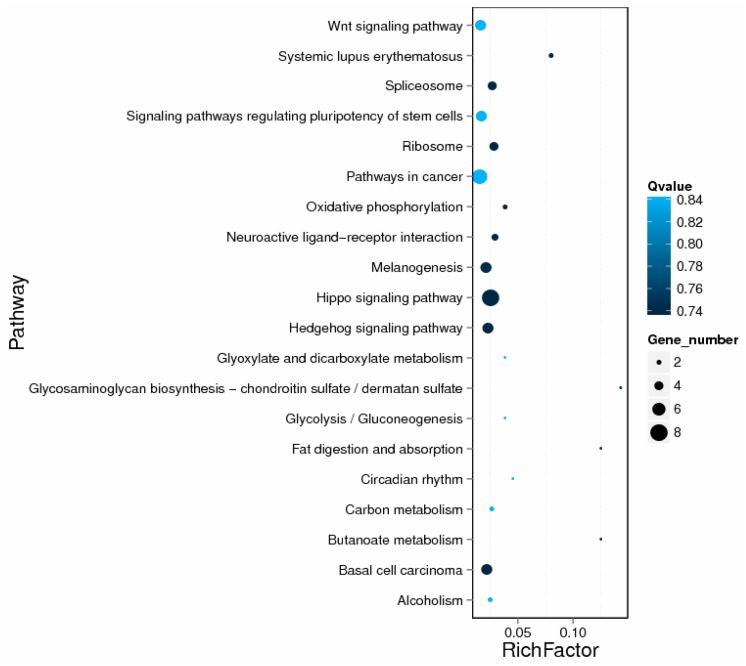
Scatter plot for the Kyoto Encyclopedia of Genes and Genomes (KEGG) enrichment analysis of the DE lncRNA target genes. The Rich factor is the ratio of the number of annotated DE genes to that of the annotated genes in this pathway term. The *Q*-value is the corrected *p*-value ranging from 0 to 1, and a lower Q-value indicates greater pathway enrichment Table 1. GO significant functional enrichment (*p* < 0.05) analysis of DE lncRNA target genes in activated hHSCs (con) and quiescent hHSCs (VPA).

Gene ontology (GO) functional enrichment analysis indicated that most of the enriched GOs were involved in 1347 biological processes, 661 cellular component and 243 molecular functions (Figure S1). The DE lncRNAs target genes were identified to be significantly enriched for five functional terms, of which four terms, such as nucleosome, DNA bending complex, DNA packaging complex and actin cap, were enriched from the cellular component ontology, one term, growth factor binding, was from the function ontology (Table 1). Kyoto Encyclopedia of Genes and Genomes (KEGG) analysis demonstrated that the significantly (*p* < 0.05) enriched pathways in the DE lncRNAs target genes correspond to the Hippo signaling pathway (Figure 5 and Table S3). In this study, some DE lncRNAs regulated hHSCs activation by participating in nucleosome, DNA bending/packaging complex, chromatin assembly, growth factor binding and the Hippo signaling pathway. However, these results also require further validation.

### 2.6. Co-Expression Analysis between Differentially Expression (DE) lncRNAs–mRNAs

**Figure 6:**
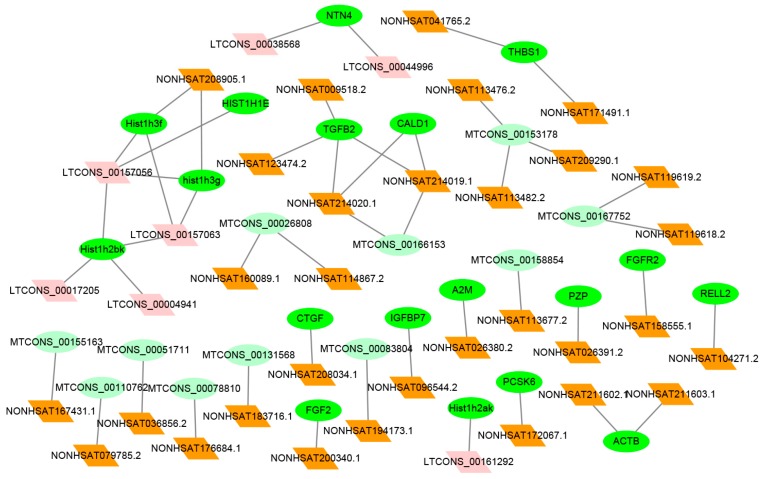
Co-expression network for the DE lncRNAs-mRNAs. A total of 38 DE lncRNAs and 29 mRNA from GO and KEGG (significant enrichment, *p* < 0.05) were used for co-expression analysis by the Cytoscape software. Known/novel mRNA are shown in green/light green and known/novel lncRNA are shown in brown/pink.

Co-expression network analysis demonstrated 38 DE lncRNAs target 30 mRNAs, including 11 novel mRNAs (light green), and each lncRNA was co-expressed with one or multiple mRNAs (Figure 6). The *CTGF*, *FGF2* and *NTN4* were co-expressed with NONHSAT208034.1, NONHSAT200340.1 and two lncRNAs, including LTCONS_00038568 and LTCONS_00044996. The top 10 regulated mRNAs and their co-expressed lncRNAs were listed in Table 2. Additionally, other additional information (fold differences; location) for co-expressed lncRNAs and mRNAs were listed in Table S3.

### 2.7. Verification of DE lncRNA and Co-Expression mRNA

**Figure 7:**
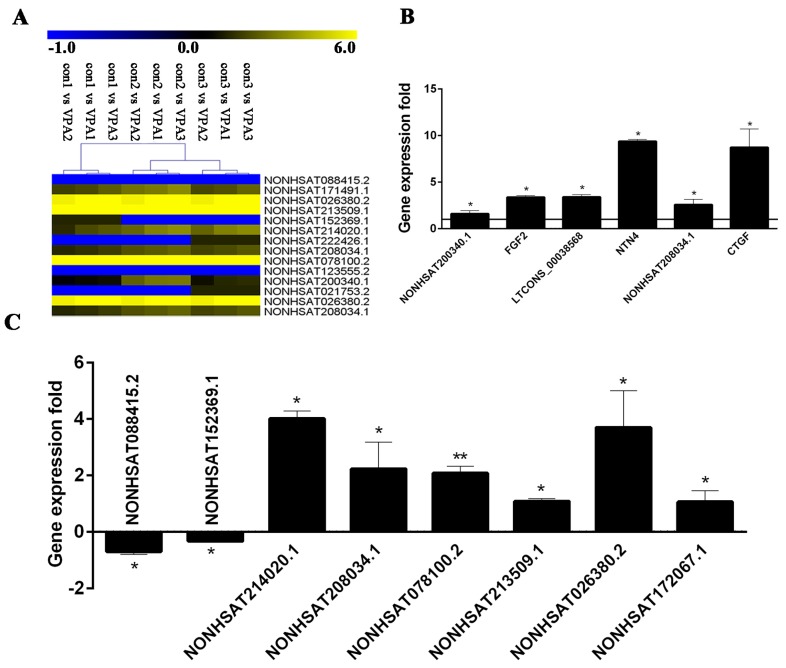
Verification of RNA-seq data. Heat maps show the differential expression fold (log2Ratio) for eight DE lncRNAs and three pairs of co-expressed lncRNAs-mRNAs between con (activated hHSC) and VPA (quiescent hHSC). Blue represents downregulation, whereas yellow indicates upregulation (**A**). The sequencing differential expression of *CTGF*, *FGF2* and NTN4 is shown in Table 2. The expression folds of eight DE lncRNAs and three pairs of co-expressed lncRNAs-mRNAs in VPA (quiescent hHSC) were normalized against con (activated hHSC) (**B**,**C**). The relative expression levels were normalized to the expression amount of glyceraldehyde-3-phosphate dehydrogenase (*GAPDH*); (* *p* < 0.05, ** *p* < 0.01).

A total of eight DE lncRNAs and three pairs of co-expressed lncRNA–mRNA were randomly selected for the validation of the relative expression levels in hHSC myofibroblasts in the con (activated hHSC) and VPA (quiescent hHSC) groups by using quantitative Real-Time Polymerase Chain Reaction (qRT-PCR). Results demonstrated that all differential expression levels of the selected lncRNAs and co-expressed lncRNAs-mRNA were consistent with the RNA-seq results (Figure 7A–C). Furthermore, one of the observation is that the expression levels of *FGF2* and *NTN4* increased in VPA (quiescent hHSC) and consistent with the previous report [8], which suggested that their targeted lncRNAs likely have these function in hepatic fibrosis.

## 3. Discussion

VPA defines a novel class of histone deacetylase (HDAC) inhibitor, which can inhibit HSC activation in vitro and in vivo [25,26]. Thus, we used VPA to induce hHSC myofibroblasts into inactive hHSCs. Immunofluorescence and qRT-PCR were employed to quantify the expression of hepatic-fibrosis-related mRNA, including α-SMA, *ACTA2*, *COL1A*, *LOX* and *LOXL2*. The results showed that VPA effectively reversed hHSCs activation and played an important role in anti-hepatic fibrosis, and successfully established a quiescence hHSCs model, which provided the conditions for downstream screening of anti-fibrosis associated lncRNA in hHSCs. lncRNAs have been paid close attention as novel regulatory players in cellular and biological processes over the past few years through high-throughput transcriptome analysis [14,15,16,17,18,19,20,21,22,23,24,25,26,27]. However, as previously described, few studies investigated lncRNAs in HSCs, especially hHSCs. Numerous protein-coding genes that play a key regulatory role in human HSC function have been reported. Even so, the diversity of lncRNA expression and its biological function in hHSCs remain unclear. In this study, we applied RNA-seq analysis to identify the lncRNA expression profile changes in hHSC myofibroblasts and examined the possible function of DE lncRNAs between activated hHSC (con) and quiescent hHSC (VPA).

In the sequencing data, the expression levels of the lncRNAs were higher than those of mRNAs (Figure S2). These findings indicated that these the lncRNAs expression in hHSC myofibroblasts may have a more critical regulatory role than mRNA expression, so we focused on lncRNA expression.

A total of 34,146 DE lncRNAs were obtained in hHSCs activation and quiescence, only 3763 DE lncRNAs and 1685 DE mRNAs were statistically significant and the results similar to previous study [21], the demonstrated that VPA-induced hHSCs quiescence has a dramatic effect on the expression profile of lncRNA. However, VPA as one of HDAC inhibitor to reverse the activation of hHSC and play an anti-liver fibrosis, thus, one part of 34,146 DE lncRNAs expression profiles were directly regulated by histone deacetylation, while another were indirectly modified by histone deacetylation. However, both the changes in lncRNA expression profiles, either directly or indirectly modified by histone acetylation was an internal mechanism of anti-fibrotic effect induced by VPA-induced hHSCs activation. We performed GO and KEGG analyses to gain insight into the characterization of the molecular functions and pathway for the target genes of DE lncRNAs [28], and predict the possible biological functions and fibrosis regulation mechanisms of target genes of DE lncRNAs between con (activated hHSC) and VPA (quiescent hHSC). GO analysis showed that the DE lncRNAs targeted genes, such as histone cluster 1 H2B family (*Hist1h2bk*), histone cluster 1 H3 family member g (hist1h3g) and histone cluster 1, H3f (*Hist1h3f*) were significantly enriched in the GO terms involving nucleosome, DNA bending complex, DNA packaging complex and DNA packaging complex term. In the functional ontology, the target genes of DE lncRNAs, namely, the upregulated genes, such as connective tissue growth factor (*CTGF*), and fibroblast growth factor 2 (*FGF2*) were significantly associated with the growth factor binding term. Furthermore, the KEGG analysis indicated that some target genes were significantly associated with the Hippo signaling pathway. These results were closely related to previous reports, for example, *FGF2* is a key factor inducing α-SMA expression changes in hHSCs and primary HSCs. Moreover, in a bile-duct-ligated mouse study, *FGF2* administration also ameliorated hepatic fibrosis and significantly reduced HSC activation [8]. Hippo pathway and its transcription factor *YAP* were thought to be key pathways in the activation HSCs in mice liver fibrosis by CCl4 administration [29]. In addition, the Hippo pathway also regulated liver organ size [11]. Hence, it is significant to identify the lncRNAs that regulate these genes as well as to study the Hippo pathway.

An lncRNAs-mRNA co-expression network was further constructed to show the potential involvement of hHSC-activated lncRNAs and mRNAs. We selected lncRNAs target genes from significantly enriched ontologies and pathways in GO and KEGG to construct an lncRNAs-mRNA co-expression network. In this network, the lncRNAs may be involved in hepatic fibrosis development by regulating the activation and quiescence of hHSCs, because their co-expressed mRNAs exert a significantly regulatory effect on hHSCs activation and quiescence. Furthermore, several lncRNAs target genes that have been reported to associated with HSC activated and liver fibrosis, such as *CTGF*, *FGF2* [8,9], *FGFR1* [30], *IGFBP7* [31] and *NTN4* [32]. Previous studies have shown that *CTGF* is associated with inflammation, pathogenesis and progression of hepatic fibrosis [33], in the Hippo signaling pathway and inhibition of MST1/2 signaling. *CTGF* was up-regulated in HepG2 cells, thereby improving tissue damage and repair [34]. Another study elucidated that *FGF2* triggered rapid phosphorylation of c-Jun N-terminal kinase (JNK) and c-JUN. Reduced a-SMA expression significantly reduces HSC activation for improvement of liver fibrosis [8], which indicated that the co-expression lncRNA NONHSAT208034.1 and NONHSAT200340.1 likely have these functions by targeting *CTGF* and *FGF2*. Netrin-4 (*NTN4*) was reduced in breast cancer tissues, whereas over-expression of *NTN4* decreased the expression of N-cadherin and vimentin, ultimately hindering epithelial-mesenchymal transition (EMT) [32], although this process was confirmed in the breast cancer mechanism, however, the *NTN4* was significantly up-regulated in quiescent hHSC (VPA) group (*p* < 0.05) in our data (Table S4). Therefore, we proposed they co-expression of LTCONS_00038568 and LTCONS_00044996 may regulated the expression of *NTN4* and attenuated hHSC activation via EMT. These lncRNAs show that the network can be meaningful for hHSC activation and quiescence or liver damage repair and regeneration. However, the results require further studies for confirmation.

Moreover, eight DE lncRNAs and three pairs of co-expressed lncRNAs-mRNA were randomly selected for the validation of the relative expression levels in hHSC myofibroblasts between activated hHSCs and quiescent hHSCs. Basing on the results of GO analysis and previous reports, we speculated that the genes that were enriched to growth factor binding terms, such as *FGF2*, *NTN4* and *CTGF* and their co-expressed lncRNAs in this study may be closely related to hHSC activation and liver injury.

In summary, we analyzed the expression profiles of lncRNA in hHSC myofibroblasts to gain insight into their critical regulatory roles in hHSC activation and quiescence and hepatic fibrosis development. Our study may help identify the key lncRNAs that potentially affect hHSC activation and quiescence or hepatic fibrosis development, as well as their possible regulatory mechanisms. For example, NONHSAT200340.1 may targeted *FGF2* to regulate activation of hHSCs via JNK signaling. LTCONS_00038568 also possible to target *NTN4* to improve liver fibrosis by reversal of EMT. Further study of these lncRNAs can provide useful insights into and new research directions for hepatic fibrosis.

This study focused on the identification of key lncRNAs in hHSCs myofibroblasts, but also understanding the role of these lncRNAs in activation of hHSCs and hepatic fibrosis. However, these lncRNAs also need to be validated in combination with animal models and patient tissue. Previous study suggested that HSCs are considered a major contributor to the tumor microenvironment in hepatocellular carcinoma (HCC), and activated HSCs promote the growth of hepatocellular carcinoma in subcutaneous xenograft model [35]. Therefore, this study not only applies to liver fibrosis molecular therapy, drug targets and biomarkers, but also can be applied to lncRNA regulation of hepatic stellate cell in xenograft research.

## 4. Materials and Methods

### 4.1. Cell Culture and Treatments

The hHSCs used were purchased from ScienCell and grown in Dulbecco’s modified Eagle medium (DMEM; Gibico, Beijing, China) supplemented with 5% fetal bovine serum (FBS; Hyclone, Melbourne, Australia) and 1% penicillin/streptomycin (P/S; Hyclone). The stock solution of valproic acid (VPA; Sigma-aldrich, Laramie, WY, USA) was prepared in dimethyl sulfoxide (DMSO) and sterilized by filtration through a 0.22-Am filter followed by the preparation of 1.25, 2.5 and 5.0 mM VPA with DMEM (5% FBS; 1% P/S). Induction of the static-like phenotype was performed by treating hHSCs with VPA-containing DMEM (5% FBS; 1% P/S) for 6 h and 4 days. Cellular immunofluorescence, qRT-PCR analysis and RNA sequencing (RNA-seq) were performed 4 days after the 5.0 mM VPA treatment of hHSCs.

### 4.2. Immunofluorescence

The hHSCs were grown in 12-well plates and treated with DMEM (5% FBA; 1% P/S) in the absence or presence of 5.0 mM VPA for 6 h and 4 days. The cells were washed with PBS buffer and incubated in 3% sheep serum at room temperature for 1 h, Next, α-SMA primary antibody (rabbit, diluted 1:100, ab5694; Abcam, Cambridge, UK) overnight at 4 °C, and then washed with PBS buffer. Secondary antibody (goat anti-rabbit, diluted 1:250, A11008; Life Technologies, Carlsbad, CA, USA, Alexa Fluor@488) was added to the wells and incubated 20 min at room temperature. Finally the pictures of the cells were obtained with a ZOETM Fluorescent Cell Inager (BIO RAD, 742BR1669, Singapore).

### 4.3. Quantitative Real-Time PCR

The total RNA was extracted from hHSCs using a QuickGene RNA cultured cell kit S (RC-S, Osaka, Japan) in accordance with the instructions of the manufacturers. Quantitative real-time PCR was performed on Bio-Rad CFX96 with a SuperReal PreMix Plus Kit (TIANGEN, Beijing, China), and the relative expression of mRNAs and lncRNAs were analyzed through the 2^−ΔΔ*C*t^ method. The mRNA and lncRNA primers are shown in Table 3.

### 4.4. cDNA Library Construction and Sequencing

Two groups of six sample cDNA libraries for activated hHSCs (con) and quiescent hHSCs (VPA, 5.0 mM 4 days) (each group with three biological repetitions) were constructed and sequenced using the Illumina HiSeq 2000 Platform. Total RNA was isolated from the hHSCs (con and VPA) and purified using the Trizol reagent. Subsequently the quality and quantity of the purified RNA samples were assessed by using an Agilent 2100 Bioanalyzer. The RNA samples with high purity (28S/18S > 1.9) and high integrity (RIN > 7.8) were employed for cDNA library construction. Isolated RNA was ribo-depleted using the Ribo-Zero™ rRNA Removal Kit (Epicentre, Madison, WI, USA) in accordance with the manufacturer’s instructions. RNA was fragmented at a temperature and in an ionic environment after purification. The short fragment RNA was reverse transcribed to synthesize first strand cDNA by using random primers and reverse transcriptase of the TruSeq^®^ Stranded kit. Then double-strained cDNA was synthesized by using DNA polymerase I and RNase H. In the cDNA two-strand synthesis process, the RNA template was removed, and dTTP was replaced by dUTP. The double-stranded cDNA product was then subjected to an ‘A’ base and a linker. We ligation product was amplified and purified to obtain the final cDNA library. Finally, the library products were prepared for Illumina HiSeq2000 sequencing (Beijing Genomics Institute (BGI), Wuhan, China).

### 4.5. Alignment and Transcript Assembly

Clean reads were mapped to the human reference genome (Hg19) using HISAT v2.0.4 [36] (http://www.ccb.jhu.edu/software/hisat).

We then used StringTie v1.0.4 [37] (http://ccb.jhu.edu/software/stringtie) to assemble transcripts through the following strategy

1. Reads were splice aligned to the genome.
2. A graph representing alternative splicing events was constructed.
3. The graph were aligned transversely to assemble.
4. Isoforms were assembled.

The new transcripts were compared with known mRNAs and lncRNAs using Cuffcompare v2.2.1 (http://cole-trapnell-lab.github.io/cufflinks). Then, the assembled single transcript was filtered and reserved through the parameterization of the transcripts with fragments per kilobase of exon per million fragments mapped (FPKM) of ≥0.5; Coverage of >1; Length of >200; and i, j, u, x, o five types of transcripts.

### 4.6. Identification of lncRNAs and mRNAs

The mRNAs or lncRNAs in the assembled transcripts were identified using CPC v0.9-r2 (http://CPC.cbi.pku.edu.cn), txCdsPredict (http://hgdownload.soe.ucsc.edu/admin/jksrc.zip), CNCI (https://github.com/www-bioinfo-org/CNCI) and pfam_scan.pl (http://pfam.xfam.org/) with the following threshold setting: CPC_threshold = 0 (assembled transcripts > 0 was mRNA, or lncRNA), CNCI_threshold = 0 (assembled transcripts > 0 was mRNA, or lncRNA), txCdsPredict_threshold = 500 (assembled transcripts > 0 was mRNA, or lncRNA), and Pfam as a protein database. The assembled transcripts were considered mRNAs if comparable to Pfam or lncRNAs. If not, all of the four judgment methods were consistent, and we only confirmed whether the transcripts were mRNAs or lncRNAs.

### 4.7. Differential Gene Expression Analyses

We used the method based on FPKM to calculate the expression of protein-coding and lncRNA genes, and the following equation was used:

$$FPKM=\begin{array}{cc} 10^{9} & \left(C\right) \end{array}/NL.$$

Here, *FPKM* is the expression of gene A, *C* is the number of reads uniquely aligned to gene A, *N* is the total number of reads uniquely aligned to all genes, and *L* is the number of bases in gene A. The sensitivity for detection of expression changes of protein-coding and lncRNA genes between Con and VPA was determined by employing DEGseq and possionDis. Meanwhile, “fold change ≥ 2.00 and FDR ≤ 0.001” was adopted as the threshold for the evaluation of significant differences in gene expression.

### 4.8. Gene Ontology (GO) Terms and Kyoto Encyclopaedia of Genes and Genomes (KEGG) Pathway Enrichment Analysis

GO, (http://amigo.geneontology.org/amigo/landing) and KEGG, (http://www.kegg.jp/) are two databases that annotate genes and gene products. GO analysis was conducted for the quantitative evaluation of molecular functions that were significantly enriched in DE mRNAs and adjacent genes of predicted lncRNAs. In GO enrichment analysis of functional significance, a hypergeometric test was performed for the mapping of all the DE mRNAs and DE lncRNAs that target mRNAs to terms in the GO database. In this process, significantly enriched GO terms were search in the DE mRNAs and DE lncRNAs that target mRNAs compared with the genomic background and calculated in accordance with Equation:

$$P=1-\sum_{i-1}^{m-1}\frac{\begin{array}{cc} \left(\begin{array}{c} M\\ i \end{array}\right) & \left(\begin{array}{c} N-M\\ n-i \end{array}\right) \end{array}}{\left(\begin{array}{c} N\\ n \end{array}\right)}.$$

Here, *N* is the number of all genes with GO annotation, *n* is the number of DE mRNAs and DE lncRNAs that target mRNAs in *N*, *M* is the number of all genes with annotated GO terms and *m* is the number of DE lncRNAs that target mRNAs in *M*. The calculated *p*-value was adjusted through FDR correction, and FDR ≤ 0.01 was selected as the threshold. The GO terms that satisfied this condition were defined as significantly enriched GO terms in the DE mRNAs and DE lncRNAs that target mRNAs. The specific pathways significantly enriched in DE mRNAs and lncRNAs with their target genes were then determined. KEGG analysis was performed for the identification of the biological pathways significantly enriched in the target genes of DE lncRNAs, and the formula was the same as that in the GO enrichment analysis. *N* is the number of all genes with KEGG annotation, *n* is the number of DE mRNAs and DE lncRNAs that target mRNAs in *N*, *M* is the number of all genes annotated to specific pathways, and *m* is the number of DE mRNAs and DE lncRNAs that target mRNAs in *M*. Pathways with *Q* ≤ 0.05 were considered significantly enriched.

### 4.9. Co-Expression Network Analysis and Prediction of the Target Genes of DE lncRNAs

Distinct fibrosis lncRNAs in hHSC activation were identified by the co-expression network analysis of DE lncRNAs and their target mRNAs. In the process, Pearson correlation coefficients [38] and the *p*-value (*p* < 0.05) between multiple genes were calculated. In this study, DE lncRNAs and their target mRNAs significantly enriched in the nucleosome, DNA bending/packaging complex, chromatin assembly (GO) and Hippo signaling pathway (KEGG) were selected to construct a co-expression network by using the cytoscape software. The target mRNAs of the DE lncRNAs were predicted through *cis*- and *trans*-actions.

### 4.10. Statistical Analyses

All results were expressed as mean ± standard deviation. All statistical analyses were performed using Student’s *t* test for the comparison of two groups. Differences at *p* < 0.05 were considered statistically significant.

## Figures and Tables

**Table 1:** Gene ontology (GO) significant functional enrichment (*p* < 0.05) analysis of DE lncRNA target genes in activated hHSCs (con) and quiescent hHSCs (VPA).

Gene Ontology Term	Cluster Frequency	Genome Frequency of Use	Corrected *p*-Value
Nucleosome	6/147	107/34,590	0.00108
DNA bending complex	6/147	107/34,590	0.00108
DNA packaging complex	6/147	124/34,590	0.00253
Actin cap	3/147	13/34,590	0.00335
Growth factor binding	8/135	258/31,397	0.00334

Cluster frequency: the denominator represents the total number of genes with GO annotation, and the numerator represents the number of each GO term gene. Genome frequency of use: the denominator represents the number of reference genes with GO annotation, and the numerator represents the number of reference genes annotated in the listed GO term. Corrected *p*-value: the *p*-value in the hypergeometric test after correction.

**Table 2:** The list of top 10 regulatory genes and their co-expressed lncRNA. The correlation (COR) represented Pearson-correlation.

Gene Symbol	Up-/Downregulated	Chromosomal Location	Co-Expressed lncRNA	Up-/Downregulated	Chromosomal Location	COR	*p*-Value
*CTGF*	up	chr6	*NONHSAT208034.1*	up	chr6	0.8812	6.19 × 10^−136^
*FGF2*	up	chr6	NONHSAT200340.1	up	Chr4	0.9053	1.11 × 10^−12^
*NTN4*	up	chr12	LTCONS_00038568	up	Chr12	0.8797	1.15 × 10^−14^
			LTCONS_00044996	up	chr12	0.9053	1.43 × 10^−51^
*FGFR2*	up	chr10	NONHSAT158555.1	up	Chr10	0.7655	0.000175775
*IGFBP7*	up	chr4	NONHSAT096544.2	up	Chr4	0.956	0.00334
*PCSK6*	up	chr15	NONHSAT172067.1	up	Chr15	0.6457	2.36 × 10^−5^
*Hist1h2ak*	down	chr6	LTCONS_00161292	up	chr6	−0.6806	0.000102578
*Hist1h3f*	down	chr6	LTCONS_00157063	down	chr6	0.9388	1.04 × 10^−158^
*CALD1*	down	chr7	NONHSAT214019.1	up	chr7	−0.6686	5.21 × 10^−7^
*hist1h3g*	down	chr6	LTCONS_00157063	down	chr6	0.8589	1.04 × 10^−158^

**Table 3:** The primer sequences used in this study.

Gene Symbol	Primer Sequence	Product Size (bp)
18S-F	AGGTGGAACGTGTGATCACC	146
18S-R	CAGGTCTTCACGGAGCTTGT	
ACTA2-F	ACTGGGACGACATGGAAAAG	129
ACTA2-R	GAGTCATTTTCTCCCGGTTG	
COL1A1-F	TTCTGCAACATGGAGACTGG	134
COL1A1-R	AATCCATCGGTCATGCTCTC	
LOX-F	GACCTGCTTGATGCCAACAC	126
LOX-R	TCCCTGTGTGTGTGCAGTAC	
LOXL2-F	ATGGGCTTGCAGAAGAAGCT	116
LOXL2-R	GTTTTGGCCACACACCATCC	
GAPDH-F	AATCCCATCACCATCTTCCA	120
GAPDH-R	TGGACTCCACGACGTACTCA	
CTGF-F	CAGCATGGACGTTCGTCTG	115
CTGF-R	AACCACGGTTTGGTCCTTGG	
FGF2-F	CCGTTACCTGGCTATGAAGG	144
FGF2-R	TTCAGTGCCACATACCAACTG	
NTN4-F	ATGCTTGCAAACCGTGTTCC	137
NTN4-R	CATGCACCTGTCACAACGTC	
NHSAT088415.2-F	GTTCGGACAAGAGCCAGGA	120
NHSAT088415.2-R	CCAACTGCCAAGTTCCTTCC	
TCONS_00038568-F	GTGTCCAGAGCAGTGCTTCT	149
TCONS_00038568-R	TAATGCAGAGACCCAGGCC	
NONHSAT208034.1-F	CCCACAGGTCTTGGAACAGG	132
NONHSAT208034.1-R	AAAGCGGTGTTTGTGTGCTC	
NONHSAT152369.1-F	CAGGTGAATGCCACACAGGT	132
NONHSAT152369.1-R	TGCTGACTGAGGATGAGATGG	
NONHSAT214020.1-F	GTGATCAGGAGGAGCTCTTGT	113
NONHSAT214020.1-R	CAGGTAGCATTGACTCCCGT	
NONHSAT078100.2-F	TCTCCAGGCTCCACAATACC	119
NONHSAT078100.2-R	TGTTCATTGCCTGCTCTCAC	
NONHSAT213509.1-F	ATGAGGGAATCCCCAATTTC	120
NONHSAT213509.1-R	AGCTGTGGATGCTTTCTGCT	
NONHSAT026380.2-F	TCTCCAGGCTCCACAATACC	120
NONHSAT026380.2-R	GACACTCCAAATCCCAGGAA	
NONHSAT172067.1-F	ACAGATGCTCAGCGATGTTG	127
NONHSAT172067.1-R	AGGCAGGAGGATCTGGATTT	
NONHSAT200340.1-F	CCCAGATGATGGCTACTGGT	142
NONHSAT200340.1-R	CCCACCTCAACACCAAAGAT	

## Supplementary Materials

Supplementary materials can be found at http://www.mdpi.com/1422-0067/19/3/675/s1.

## Abbreviations

hHSC	human hepatic stellate cell
lncRNA	Long noncoding RNA
RNA-seq	RNA sequencing
DE	differentially expressed
GO	Gene Ontology
KEGG	Kyoto Encyclopaedia of Genes & Genomes
CTGF	connective tissue growth factor
FGF2	fibroblast growth factor
NTN4	netrin-4
ACTA2	actin, alpha2 smooth muscle
COL1A1	collagen type I alpha 1 chain
LOX	lysyl oxidase
LOXL2	lysyl oxidase like 2
FGFR2	fibroblast growth factor receptor 2
TGF-β	transforming growth factor beta
PPAR	peroxisome proliferator-activated receptors
ChIP-seq	Chromatin immunoprecipitation sequencing
α-SMA	actin, aortic smooth muscle
HDAC	Histone Deacetylase
IGFBP7	insulin like growth factor binding protein 7
FPKM	fragments per kilobase of exon per million fragments mapped
RPKM	Reads Per Kilobase per Million mapped reads

## References

[1] Higashi T., Friedman S.L., Hoshida Y. (2017). Hepatic stellate cells as key target in liver fibrosis. Adv. Drug Deliv. Rev..

[2] Huang Y., Deng X., Liang J. (2017). Modulation of hepatic stellate cells and reversibility of hepatic fibrosis. Exp. Cell Res..

[3] Schmitt-Graff A., Kruger S., Bochard F., Gabbiani G., Denk H. (1991). Modulation of alpha smooth muscle actin and desmin expression in perisinusoidal cells of normal and diseased human livers. Am. J. Pathol..

[4] Yum M.J., Koppula S., Kim J.S., Shin G.M., Chae Y.J., Yoon T., Chun C.S., Lee J.D., Song M. (2017). Protective effects of ampelopsis brevipedunculata against in vitro hepatic stellate cells system and thioacetamide-induced liver fibrosis rat model. Pharm. Biol..

[5] Perepelyuk M., Terajima M., Wang A.Y., Georges P.C., Janmey P.A., Yamauchi M., Wells R.G. (2013). Hepatic stellate cells and portal fibroblasts are the major cellular sources of collagens and lysyl oxidases in normal liver and early after injury. Am. J. Physiol. Gastrointest. Liver Physiol..

[6] Liu X., Xu J., Brenner D.A., Kisseleva T. (2013). Reversibility of liver fibrosis and inactivation of fibrogenic myofibroblasts. Curr. Pathobiol. Rep..

[7] Ganai A.A., Husain M. (2017). Genistein attenuates d-galn induced liver fibrosis/chronic liver damage in rats by blocking the TGF-β/smad signaling pathways. Chem. Biol. Interact..

[8] Sato-Matsubara M., Matsubara T., Daikoku A., Okina Y., Longato L., Rombouts K., Thuy L.T.T., Adachi J., Tomonaga T., Ikeda K. (2017). Fibroblast growth factor 2 (*FGF2*) regulates cytoglobin expression and activation of human hepatic stellate cells via JNK signaling. J. Biol. Chem..

[9] Bohm F., Speicher T., Hellerbrand C., Dickson C., Partanen J.M., Ornitz D.M., Werner S. (2010). *FGF* receptors 1 and 2 control chemically induced injury and compound detoxification in regenerating livers of mice. Gastroenterology.

[10] LaQuaglia M.J., Grijalva J.L., Mueller K.A., Perez-Atayde A.R., Kim H.B., Sadri-Vakili G., Vakili K. (2016). *Yap* subcellular localization and hippo pathway transcriptome analysis in pediatric hepatocellular carcinoma. Sci. Rep..

[11] Patel S.H., Camargo F.D., Yimlamai D. (2017). Hippo signaling in the liver regulates organ size, cell fate, and carcinogenesis. Gastroenterology.

[12] Zhubanchaliyev A., Temirbekuly A., Kongrtay K., Wanshura L.C., Kunz J. (2016). Targeting mechanotransduction at the transcriptional level: *Yap* and brd4 are novel therapeutic targets for the reversal of liver fibrosis. Front. Pharmacol..

[13] Guttman M., Amit I., Garber M., French C., Lin M.F., Feldser D., Huarte M., Zuk O., Carey B.W., Cassady J.P. (2009). Chromatin signature reveals over a thousand highly conserved large non-coding RNAs in mammals. Nature.

[14] Krzyzanowski P.M., Muro E.M., Andrade-Navarro M.A. (2012). Computational approaches to discovering noncoding RNA. RNA.

[15] Fu L.L., Li C.J., Xu Y., Li L.Y., Zhou X., Li D.D., Chen S.X., Wang F.G., Zhang X.Y., Zheng L.W. (2017). Role of lncRNAs as novel biomarkers and therapeutic targets in ovarian cancer. Crit. Rev. Eukaryotic Gene Express..

[16] Greene J., Baird A.M., Brady L., Lim M., Gray S.G., McDermott R., Finn S.P. (2017). Circular RNAs: Biogenesis, function and role in human diseases. Front. Mol. Biosci..

[17] Lavorgna G., Vago R., Sarmini M., Montorsi F., Salonia A., Bellone M. (2016). Long non-coding RNAs as novel therapeutic targets in cancer. Pharmacol. Res..

[18] Zhang Y., Yang H., Han L., Li F., Zhang T., Pang J., Feng X., Ren C., Mao S., Wang F. (2017). Long noncoding RNA expression profile changes associated with dietary energy in the sheep testis during sexual maturation. Sci. Rep..

[19] Yu-Wai-Man C., Owen N., Lees J., Tagalakis A., Hart S., Webster A., Orengo C., Khaw P. (2017). Genome-wide RNA-sequencing analysis identifies a distinct fibrosis gene signature in the conjunctiva after glaucoma surgery. Sci Rep.

[20] Guo C.J., Xiao X., Sheng L., Chen L., Zhong W., Li H., Hua J., Ma X. (2017). RNA sequencing and bioinformatics analysis implicate the regulatory role of a long noncoding RNA-mRNA network in hepatic stellate cell activation. Cell Physiol. Biochem..

[21] Zhou C., York S.R., Chen J.Y., Pondick J.V., Motola D.L., Chung R.T., Mullen A.C. (2016). Long noncoding RNAs expressed in human hepatic stellate cells form networks with extracellular matrix proteins. Genome Med..

[22] Yu F., Jiang Z., Chen B., Dong P., Zheng J. (2017). Neat1 accelerates the progression of liver fibrosis via regulation of microRNA-122 and kruppel-like factor 6. J. Mol. Med..

[23] Zhang K., Han X., Zhang Z., Zheng L., Hu Z., Yao Q., Cui H., Shu G., Si M., Li C. (2017). The liver-enriched *Lnc-LFAR1* promotes liver fibrosis by activating tgfβ and notch pathways. Nat. Commun..

[24] Nawrocki E.P., Kolbe D.L., Eddy S.R. (2009). Infernal 1.0: Inference of RNA alignments. Bioinformatics.

[25] Gao F., Shen X.Z., Jiang F., Wu Y., Han C. (2016). DNA-guided genome editing using the Natronobacterium gregoryi Argonaute. Nat. Biotechnol..

[26] Gottlicher M., Minucci S., Zhu P., Kramer O.H., Schimpf A., Giavara S., Sleeman J.P., Coco F.L., Nervi C., Pelicci P.G. (2001). Valproic acid defines a novel class of HDAC inhibitors inducing differentiation of transformed cells. EMBO J..

[27] Wang P., Xue Y., Han Y., Lin L., Wu C., Xu S., Jiang Z., Xu J., Liu Q., Cao X. (2014). The STAT3-binding long noncoding RNA *LNC-DC* controls human dendritic cell differentiation. Science.

[28] Conesa A., Madrigal P., Tarazona S., Gomez-Cabrero D., Cervera A., McPherson A., Szczesniak M.W., Gaffney D.J., Elo L.L., Zhang X. (2016). A survey of best practices for RNA-seq data analysis. Genome Biol..

[29] Mannaerts I., Leite S.B., Verhulst S., Claerhout S., Eysackers N., Thoen L.F., Hoorens A., Reynaert H., Halder G., van Grunsven L.A. (2015). The hippo pathway effector yap controls mouse hepatic stellate cell activation. J. Hepatol..

[30] Lin N., Chen S., Pan W., Xu L., Hu K., Xu R. (2011). Np603, a novel and potent inhibitor of *FGFR1* tyrosine kinase, inhibits hepatic stellate cell proliferation and ameliorates hepatic fibrosis in rats. Am. J. Physiol. Cell Physiol..

[31] Sun R.F., Liu L.X., Zhang H.Y. (2009). Effect of tanshinone ii on hepatic fibrosis in mice. Zhongguo Zhong Xi Yi Jie He Za Zhi.

[32] Xu X., Yan Q., Wang Y., Dong X. (2017). *NTN4* is associated with breast cancer metastasis via regulation of EMT-related biomarkers. Oncol. Rep..

[33] George J., Tsutsumi M., Tsuchishima M. (2017). *MMP-13* deletion decreases profibrogenic molecules and attenuates *N*-nitrosodimethylamine-induced liver injury and fibrosis in mice. J. Cell Mol. Med..

[34] Fan F., He Z., Kong L.L., Chen Q., Yuan Q., Zhang S., Ye J., Liu H., Sun X., Geng J. (2016). Pharmacological targeting of kinases MST1 and MST2 augments tissue repair and regeneration. Sci. Transl. Med..

[35] Geng Z.-M., Li Q.-H., Li W.-Z., Zheng J.-B., Shah V. (2014). Activated human hepatic stellate cells promote growth of human hepatocellular carcinoma in a subcutaneous xenograft nude mouse model. Cell Biochem. Biophys..

[36] Kim D., Langmead B., Salzberg S.L. (2015). Hisat: A fast spliced aligner with low memory requirements. Nat. Methods.

[37] Pertea M., Pertea G.M., Antonescu C.M., Chang T.C., Mendell J.T., Salzberg S.L. (2015). Stringtie enables improved reconstruction of a transcriptome from RNA-seq reads. Nat. Biotechnol..

[38] Janosky J.E. (1991). Pearson correlation coefficients vs. reliability coefficients. J. Am. Diet. Assoc..

