# Supplementary material for: Key Anti-Fibrosis Associated Long Noncoding RNAs Identified in Human Hepatic Stellate Cell via Transcriptome Sequencing Analysis
Source: Int J Mol Sci. 2018 Feb 27;19(3):675. doi: 10.3390/ijms19030675 (PMC5877536; doi:10.3390/ijms19030675)
Supplement: Supplementary file 1 [file Figure Supplementary.pdf › Figure Supplementary.pdf]

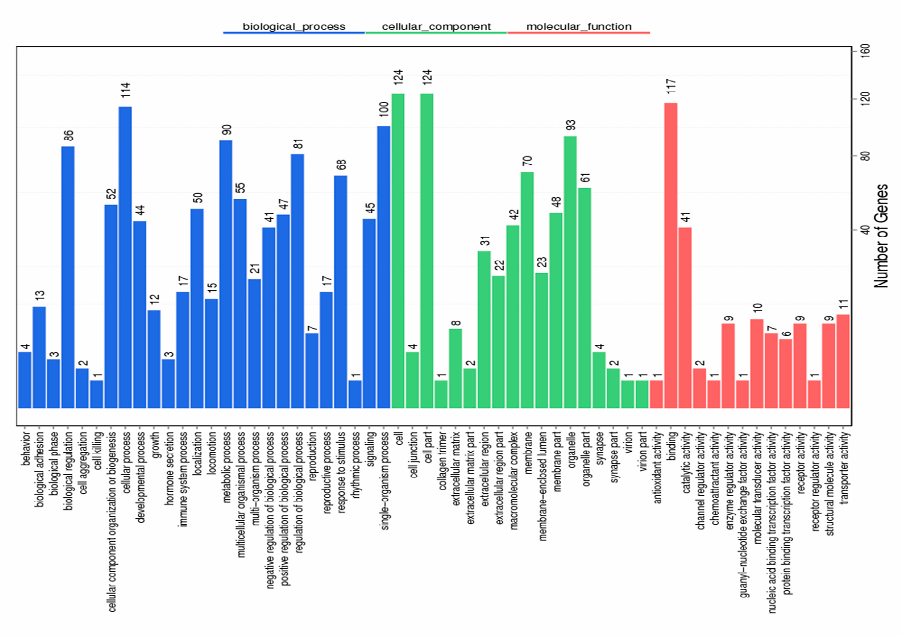

Figure S1. GO enrichment analysis of target mRNA of DE lncRNA in activated hHSCs (con) and quiescent hHSCs （VPA）. The blue, green, and red colors indicate biological processes, and cellular components, respectively.

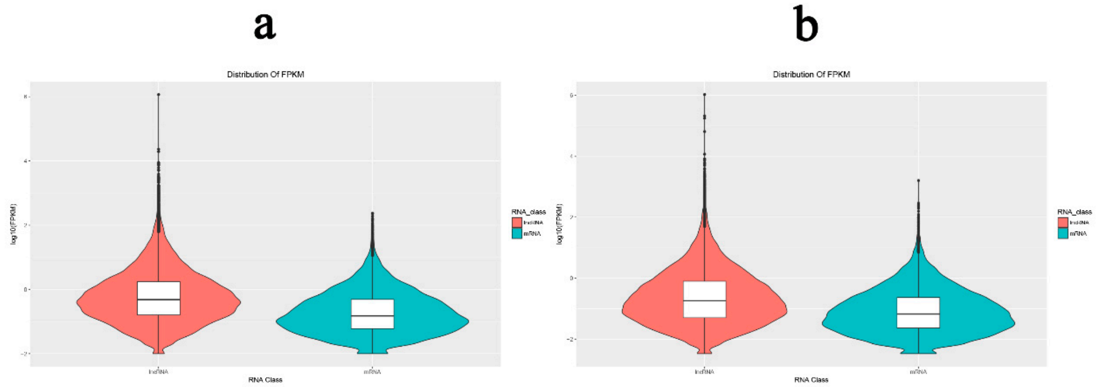

Figure S2. FPKM value of lncRNAs and mRNAs in con (hHSC myofibroblasts) and VPA (5.0mM VPA 4 day). (a and b) show the expression levels of lncRNAs and mRNAs in con (hHSC myofibroblasts) and VPA (5.0mM VPA 4 day), respectively.
