# Supplementary material for: Key Anti-Fibrosis Associated Long Noncoding RNAs Identified in Human Hepatic Stellate Cell via Transcriptome Sequencing Analysis
Source: Int J Mol Sci. 2018 Feb 27;19(3):675. doi: 10.3390/ijms19030675 (PMC5877536; doi:10.3390/ijms19030675)
Supplement: Supplementary file 1 [file ijms-19-00675-s001.zip › Table S3.pdf]

Table S3. KEGG significant pathway enrichment analysis of the DE lncRNA target genes.

Path-way ID	Name	Cluster frenqu-ency	Genome frequency of use	P-value	Q-value	Genes
ko04390	Hippo signaling pathway	8/109	314/9593	0.02635468	0.7375082	MTCONS_00108259 MTCONS_00051711 MTCONS_00078810 MTCONS_00131568 MTCONS_00153178 MTCONS_00155163 MTCONS_00110762 MTCONS_00026808

Table S3. KEGG enrichment analysis showed that the DE lncRNA-targeted mRNAs were only significantly enriched in the Hippo signalling pathway.
